# Comparison of two prepill cortisol concentrations in dogs with hypercortisolism treated with trilostane

**DOI:** 10.1186/s12917-018-1750-3

**Published:** 2018-12-27

**Authors:** Felicitas Boretti, Caterina Musella, Wanda Burkhardt, Claudia Kuemmerle-Fraune, Barbara Riond, Claudia Reusch, Nadja Sieber-Ruckstuhl

**Affiliations:** 1 Clinic for Small Animal Internal Medicine, Vetsuisse Faculty, University of Zurich, Zurich, Switzerland; 2 Clinical Laboratory, Vetsuisse Faculty, University of Zurich, Zurich, Switzerland

**Keywords:** Prepill cortisol, Canine, Monitoring, Vetoryl

## Abstract

**Background:**

The ideal method for monitoring trilostane therapy in dogs with hypercortisolism is still open to debate. Recently, determination of the pre-trilostane (prepill) cortisol concentration has been proposed to be more repeatable than either post-trilostane or post-ACTH cortisol. The aim of this study was to compare two prepill cortisol concentrations in dogs with hypercortisolism during trilostane therapy. Sixteen client-owned dogs with naturally occurring hypercortisolism were prospectively included and cortisol concentrations were measured twice, 1 h apart, before the morning trilostane dose (prepill 1 and 2 cortisol).

**Results:**

A total of 47 prepill cortisol measurement pairs were included. Compared to prepill 1, prepill 2 cortisol was higher in 15, equal in 8 and lower in 24 pairs. Group agreement between prepill 1 and 2 cortisol was 70% (moderate agreement - weighted kappa 0.55). In 30% of the pairs, group assignment was discrepant, implying a different therapeutic decision. In some dogs certain circumstances (e.g. excessive barking, difficulties during blood collection, excitement at arrival) were identified as potential factors explaining the discrepancy between prepill 1 and 2 cortisol measurements.

**Conclusions:**

In a substantial number of dogs treated with trilostane, the two prepill cortisol concentrations differed. Part of this difference might be ascribable to stressful events during test performance. When using prepill cortisol measurements to monitor trilostane therapy, recording of any incident during handling that might affect cortisol release might be helpful to make a reliable decision about a trilostane dose adaptation.

## Background

For a long time, the ACTH stimulation test has been considered the method of choice for monitoring trilostane therapy in dogs [1–6]. As trilostane is short-acting and shows its main effects a few hours after application, the results of the ACTH stimulation tests are largely dependent on the time between the last trilostane application and test administration [4, 7, 8]. Possibly overdosed dogs with baseline and post-ACTH cortisol concentrations < 2 μg/dl 3–6 h after trilostane application had a significantly higher cortisol concentration if the test was repeated 6 h later [8]. Continuing trilostane therapy without reducing the trilostane dose did not induce clinical hypoadrenocorticism and seemed safe in the majority of these dogs [8]. This raised serious concerns about the reliability of the ACTH stimulation test as a monitoring tool. In addition, in recent years, the availability of synthetic ACTH has been limited and, in some countries, a major price increase has occurred. Moreover, at high concentrations, synthetic ACTH causes adrenal damage in rats [9]. Although, low doses of ACTH (1 μg/kg) can be used in dogs receiving trilostane [10], a monitoring method not relaying on ACTH would be preferable. In several studies baseline cortisol, endogenous ACTH, cortisol/ACTH ratio, haptoglobin, urine corticoid:creatinine ratio (UCCR) and clinical signs reported by owners were evaluated, but none of them seemed convincingly superior to the ACTH stimulation test [11–16]. A recent study compared the pre-trilostane cortisol, the three-hour post-trilostane cortisol and the post-ACTH cortisol concentrations against the clinical signs reported by owners [17]. This study showed that the pre-trilostane and the three-hour post trilostane cortisol concentrations were potentially better monitoring parameters than the post-ACTH cortisol concentrations, as they differentiated better between dogs with excellent control and dogs that were inadequately controlled [17]. In addition, a further study by the same group showed that the pre-trilostane cortisol concentration is more repeatable than the post-trilostane or the post-ACTH cortisol concentration in dogs on a constant dose of trilostane [18]. Therefore, measurement of one cortisol value just before the next trilostane application seemed a potentially more reliable assessment of treatment control in trilostane-treated dogs, and one, which might replace the ACTH stimulation test.

The question remains, however, whether one single cortisol measurement in trilostane-treated dogs with hypercortisolism (HC) is sufficiently reliable. It is well known that cortisol concentrations fluctuate and are influenced by various factors, e.g. stress [19]. The agreement between two cortisol measurements taken within an hour of each other in dogs with HC and treated with trilostane, has never been evaluated. Therefore, the aim of this study was to investigate the agreement of two prepill cortisol measurements in dogs with HC during trilostane therapy.

## Results

### Comparison of prepill 1 and 2 cortisol measurements

**Fig. 1:**
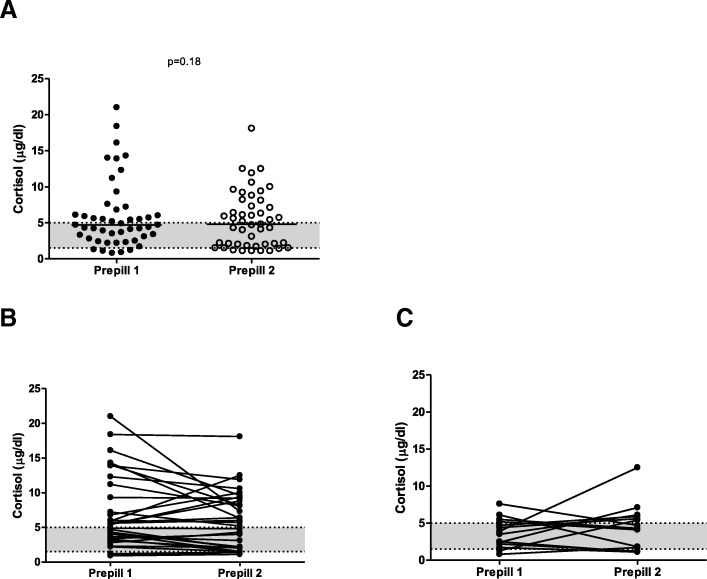
Scatter plot of prepill 1 and prepill 2 cortisol concentrations: **a** - absolute values for all cortisol pairs (*n* = 47); **b** - course of the two prepill cortisol concentrations in pairs in which both values were allocated to the same group according to the target range (1.5–5 μg/dl; *n* = 33); **c** - course of the two prepill cortisol concentrations in pairs in which the two cortisol values were not allocated to the same group (*n* = 14). The grey area represents the defined cortisol target range

A total of 47 cortisol measurement pairs from 16 dogs were included, with 13 of the dogs providing multiple pairs (2 pairs (5 dogs), 3 pairs (3 dogs), 4 pairs (1 dog), 5 pairs (3 dogs), 6 pairs (1 dog)). The length of trilostane therapy ranged from 2 to 36 weeks (median: 12). Prepill 1 cortisol ranged from 0.8 to 21 μg/dl (median: 4.7) and prepill 2 cortisol from 1.1 to 18.1 μg/dl (median: 4.8) (*p* = 0.18, 1-β = 0.2). (Fig. 1a). Compared to prepill 1 cortisol, prepill 2 cortisol was higher in 15 pairs, equal in 8 pairs and lower in 24 pairs (Fig. 1b and c).

### Group agreement

Group agreement between prepill 1 and prepill 2 cortisol was observed in 33/47 (70%) pairs and disagreement in 14/47 (30%) pairs (moderate agreement, weighted kappa 0.55 [20]) (Table 1).

**Table 1:** Group agreement between prepill 1 and 2 cortisol concentrations

	Prepill 2 cortisol (< 1.5 μg/dl)	Prepill 2 cortisol (1.5–5 μg/dl)	Prepill 2 cortisol (> 5 μg/dl)
Prepill 1 cortisol (< 1.5 μg/dl)	3	1	1
Prepill 1 cortisol (1.5–5 μg/dl)	3	13	5
Prepill 1 cortisol (> 5 μg/dl)	0	4	17

Weighted Kappa = 0.55

**Fig. 2:**
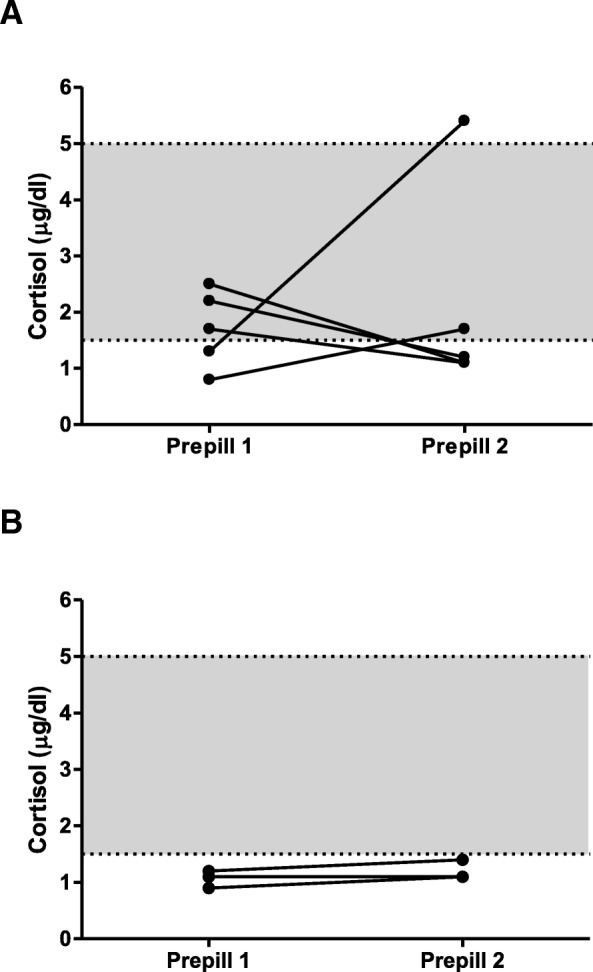
Course of prepill 1 and prepill 2 cortisol concentrations for specific cortisol pairs: **a** - all pairs in which one value was below the target range of the study. **b** - all pairs in which both values were below the target range of the study. The grey area represents the defined cortisol target range

In eight cortisol pairs at least one value was below the target range (1.5–5 μg/dl) and in 3 (37.5%) of these, both values were below the target range (Fig. 2a+b, Table 1).

In 26 cortisol pairs at least one value was within the target range and in 13 (50%) of these, both values were within the target range (Table 1).

Finally, in 27 pairs at least one cortisol value was above the target range and in 17 (63%) of these, both values were above the target range (Table 1).

### Follow-up of dogs with values below the target range

In five cortisol pairs one value was below and the second value within or above the target range (Fig. 2a). These cortisol pairs belonged to five different dogs.

In four of them, the trilostane dose was not changed, as none showed signs of hypocortisolism. All four dogs returned to our clinic after 1, 2, 3 or 6 months, respectively. At that time, none of these dogs showed prepill cortisol values below the target range. In dog five, the trilostane dose was decreased. This dog did not show clinical signs of hypocortisolism at that point, but had had three cortisol pairs with both values below the target range at the three previous visits (see below).

In three cortisol pairs, both values were below the target range (Fig. 2b). All three pairs belonged to dog five mentioned above. The dog never showed signs of hypocortisolism. The trilostane dose was gradually reduced from 1.7 mg/kg/d to 0.38 mg/kg/d. In this dog, trilostane was discontinued 6 months after the last re-evaluation included in this study. Further re-evaluations 1 and 4 months after cessation of trilostane therapy did not reveal recurrence of clinical signs of HC; the dog remained clinically healthy without any medications.

### Factors explaining differences between paired measurements

**Fig. 3:**
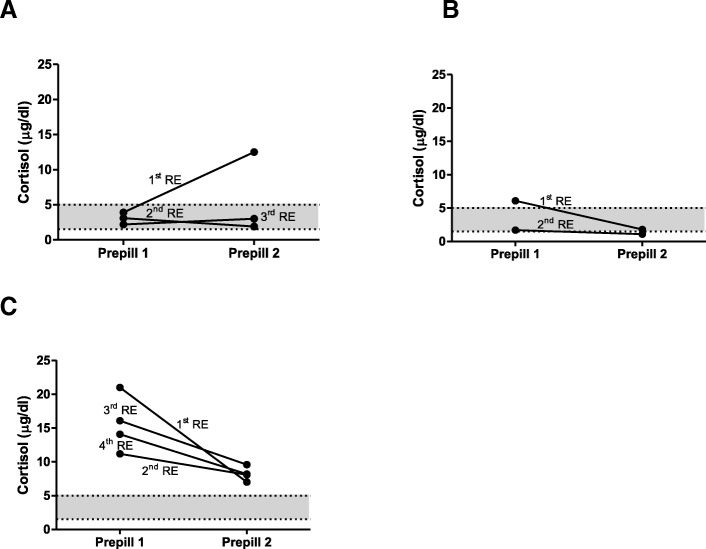
Values of prepill 1 and prepill 2 cortisol concentrations in three dogs (**a**-**c**). Dog A: stayed in the hospital at 1st re-evaluation (RE), barking the whole time. At the 2nd and 3rd RE the dog went home with owner. Constant trilostane dose of 1 mg/kg BID for all REs. Dog B: Difficulties at 1st RE in drawing blood for prepill 1; no problems drawing blood for prepill 2. No problems drawing blood at 2nd RE. Constant trilostane dose of 1 mg/kg BID for both REs. Dog C: Dog always very excited upon arrival. Stays in the hospital at each RE and calms down. Increasing trilostane doses between 1st and 4th RE (1.3–3.3 mg/kg BID). The grey area represents the defined cortisol target range

In several dogs certain circumstances (e.g. excessive barking during hospitalization, difficulties during blood collection, severe excitement during arrival) could be identified as potential factors explaining the differences between some paired prepill 1 and 2 cortisol measurements (Fig. 3).

## Discussion

The main goal of this study was to compare two prepill cortisol concentrations in dogs with HC during trilostane therapy. Although, the two cortisol values (prepill 1 and prepill 2) did not differ significantly, total group agreement between the two concentrations was only moderate, meaning that in 30% of the dogs there was a substantial difference between the two measurements. This led to a different group allocation according to the target range and possibly to differing therapeutic decisions.

Dogs with prepill cortisol concentrations below the target range are most critical, as overtreatment has to be identified and iatrogenic hypoadrenocorticism excluded. It is known that many dogs with low cortisol concentrations a few hours after trilostane application (around the time of maximal trilostane action) do not show clinical signs of hypocortisolism [8, 17]. In addition, low post-ACTH cortisol concentrations 2–3 h after trilostane application may not persist, but increase significantly when the test is repeated a few hours later and when the effect of trilostane action is tailing off [8]. In contrast, a low prepill cortisol concentration was postulated to more reliably point towards excessive suppression, as this would be about 12 h after the last dose of trilostane [17]. Our study, now, shows that even at that time point cortisol fluctuations occur, possibly leading to discrepant results. In 5 of 8 cortisol pairs with cortisol concentrations below the target range, the second value was within or even above the defined cortisol target range. We hypothesize that dogs like this are not at high risk of developing clinically relevant hypoadrenocorticism. A reduction of the trilostane dose in these dogs might lead to less control of disease due to a increase in cortisol concentrations. Follow-up of our dogs appeared to verify this hypothesis, as in 4/5 dogs the trilostane dose was not decreased and none of them developed signs of HC. If both prepill cortisol concentrations are below the target range, we hypothesize that overtreatment cannot be excluded. In this situation, the authors would opt for a dose reduction to avoid the risk of clinical hypocortisolism. In case of (subtle) clinical signs of hypocortisolism (tiredness, reduced appetite) starting glucocorticoid therapy should be considered. In three cortisol pairs in our study both values were below the target range. All three pairs belonged to the same dog. Clinically, this dog never showed signs of hypocortisolism, but the trilostane dose was gradually decreased and finally stopped. As the number of dogs included in our study is low, it is difficult to draw definitive conclusions how to proceed if discrepant results occur. The above-mentioned hypothesis, about the risk of developing hypocortisolism in dogs with low prepill cortisol values, should therefore be evaluated in further studies with a larger number of animals.

In some dogs with a substantial difference between the two cortisol values, the discrepancy could be ascribed to certain occurrences during evaluation. These events were: excessive barking during the one hour waiting time, problems drawing blood at prepill 1 or 2 cortisol measurements and severe excitement upon arrival. It has already been shown that examination and hospitalization can increase the urine cortisol to creatinine ratios [21]. Influence from environmental stress (e.g. by travelling to the hospital) has also been suggested to be a factor in altering prepill cortisol [17].

As the occurrence of such stress factors was not prospectively assessed or recorded during the study, we cannot exclude that other events occurred, possibly explaining further discrepant results. In addition, as there is no clear marker for stress in dogs, severe stress could even go unrecognized. Both values can be influenced by stress, and neither prepill 1 nor prepill 2 cortisol concentration seems clearly better than the other. However, the recording of any incident during arrival, clinical hospitalization and handling of the dog seems important to be able to later relate the values to an event and help in their interpretation.

Dogs, which have one value within the target range, but the other above the target range should also be discussed. If only the higher value had been measured, the trilostane dose would have been increased. In contrast, if only the value within the target range had been measured, the trilostane dose would not have been altered. Which treatment decision would be correct has to be evaluated in future studies.

One limitation of this study is the rather low sample size and therefore a low power of the statistical test. Therefore, our results can only be regarded as preliminary and should be confirmed in a larger study. Other limitations included the lack of prospective assessment and documentation of stressful events and the lack of a validated, standardized clinical score, to which the cortisol results can be compared. Finally, the lack of a concurrent ACTH stimulation test could also be judged as limitation. However, the goal of this study was not to assess the prepill cortisol as a monitoring tool and compare it to the post-ACTH cortisol, but to investigate the agreement between two prepill cortisol measurements.

## Conclusions

The agreement between two prepill cortisol concentrations taken one hour apart and 11–13 h after the last trilostane application is only moderate. Stressful events occurring during the re-evaluation can influence both cortisol concentrations. It is advisable to record any incident during handling of the dog, to possibly later relate the cortisol values to an event and help in their interpretation. Even though determination of two prepill cortisol values instead of one may lead to discrepant results, the additional information gained from two instead of one cortisol value can be helpful for the decision about a trilostane dose adaptation. In dogs in which both prepill cortisol concentrations are below the target range, a trilostane dose reduction should be considered.

## Methods

### Animals

Sixteen dogs with naturally occurring HC were prospectively enrolled. Eleven dogs were male (6 castrated) and 5 were female (5 spayed). Breeds included Bergamasco Sheepdog (1), Berger Blanc Suisse (1), Chihuahua (1), French Bulldog (1), Lapinkoira (1), Maltese dog (2), Petit Basset Griffon Vendéen (1), Podengo Português (1), Standard poodle (1), Yorkshire Terrier (4), and 2 mixed-breed dogs. Age ranged between 6 and 16 years (median 10) and body weight between 5 and 30 kg (median 10). The criteria for inclusion were clinical signs consistent with HC (e.g. polyuria, polydipsia, polyphagia, panting, skin problems, weakness, abdominal enlargement), a positive low-dose dexamethasone test (LDDS test) or a positive ACTH stimulation test and the agreement of the owner to treat the dog with trilostane (Vetoryl, MSD Animal Health GmbH, Lucerne, Switzerland) and to present it for regular re-evaluations. The inclusion of the dogs in the study was approved by the veterinary office of the canton of Zurich and was in accordance with the guidelines and directives established by the Animal Welfare Act of Switzerland (TVB 191/13). Written consent of all pet owners was obtained before including the dogs in the study.

### Experimental design

The prospective study was performed between September 2016 and March 2017 at the clinic for Small Animal Internal Medicine of the University of Zurich. Only dogs on twice-daily trilostane therapy were included. The starting dose of trilostane for dogs with HC was 0.5–1 mg/kg q12h. Every dog was included independent on the length of the previous trilostane therapy. At each re-evaluation, cortisol concentrations were measured twice, 1 h apart, just before the morning trilostane dose (prepill 1 and prepill 2 cortisol). Blood was drawn approximately 11–12 h after the last trilostane application for prepill 1 and 12–13 h after the last trilostane application for prepill 2. The target ranges of both cortisol concentrations were defined as 1.5–5 μg/dl [16]. Every dog was assessed by a standardized owner questionnaire and by a clinical examination. The trilostane dose was adjusted according to the prepill cortisol concentrations and the clinical signs.

For the group agreement analysis, each prepill cortisol concentration was assigned to one of three groups according to the control of cortisol release: cortisol < 1.5 μg/dl (excessive control), cortisol 1.5–5 μg/dl (adequate control) or cortisol > 5 μg/dl (inadequate control).

### Analytical procedures

Serum was stored at − 20 °C until assayed. Serum cortisol concentrations were measured by a competitive immunoassay (DPC Immulite® 2000, Siemens Schweiz AG, Zurich, Switzerland), previously validated in dogs and performed according to manufacturer’s instruction, at a commercial laboratory [22]. The accuracy of the methodologies was assessed by continuous yearly participation in an external quality assurance program, ESVE Veterinary Endocrinology External Quality Assessment Scheme (VEEEQADS). As stated by the manufacturer, the intra-assay coefficients of variation were 10 and 6% at cortisol levels of 2.7 and 18.9 μg/dl, respectively. Therefore, prepill 1 and 2 cortisol concentrations differing ≤10% were regarded as equal for the group agreement analysis by Cohen’s kappa (see below).

### Statistical analysis

Statistical analyses were performed using commercially available software (GraphPad Prism5, Graph Pad Software, San Diego, CA, USA, SPSS 24.0 for Windows, SPSS Inc., Chicago, IL USA, G^*^-Power Version 3, Heinrich Heine University, Düsseldorf, Germany). Cortisol values were tested for normality by the d’Agostino and Pearson omnibus normality test. As the data were not normally distributed, ranges and median values are reported. The Wilcoxon signed rank test was used to examine the difference between the paired measurements. The degree of agreement between group assignments was quantified by Cohen’s kappa. Differences were considered significant at *p* < 0.05.

## Abbreviations

HC: Hypercortisolism
LDDS test: Low-dose dexamethasone test
Prepill cortisol: Pre-trilostane cortisol concentration
UCCR: Urine corticoid:creatinine ratio
